# Balancing new technology: Virtual reality for balance measurement case report

**DOI:** 10.1097/MD.0000000000032799

**Published:** 2023-02-03

**Authors:** Omer Weissberger, Eran Orr, Miki Levy, Shani Kimel-Naor, Meir Plotnik, Tal Arbel

**Affiliations:** a XR Health Ltd, Tel Aviv, Israel; b XR Health Inc, Brookline, MA; c The Center of Advanced Technologies in Rehabilitation, Sheba Medical Center, Tel Hashomer, Israel; d Department of Physiology and Pharmacology, Sackler Faculty of Medicine, Tel Aviv University, Tel Aviv, Israel; e Sagol School of Neuroscience, Tel Aviv University, Tel Aviv, Israel.

**Keywords:** balance measurement, case report, Sway, virtual reality

## Abstract

Falling and the inability to maintain balance are the second leading cause of unintentional injury deaths globally. There are a number of chronic and acute conditions characterized by balance difficulties, including neurological diseases, and sport injuries. Therefore, methods to monitor and quantify balance are critical for clinical decision-making regarding risk management and balance rehabilitation. New advances in virtual reality (VR) technology has identified VR as a novel therapeutic platform. VRSway is a VR application that uses sensors attached to a virtual reality headset, and handheld remote controllers for measurement and analysis of postural stability by measuring changes in spatial location relative to the center of mass and calculates various postural stability indexes. This case report evaluates balance measures in 2 healthy participants with no previous history of balance disorders using the VRSway software application and compares to output generated by the current gold standard of balance measurement, force platform technology.

**Case Presentation:**

The primary objective of this case study was to validate the VRSway stability score for evaluation of balance. Here, we present posturography measures of the VRSway in comparison with force plate readouts in 2 healthy participants. Body Sway measurements were recorded simultaneously in both the force plate and VRSway systems. Data calculated by proprietary software is highly correlative to the data generated by force plates for each of the following measurements for participant-1 and participant-2, respectively: Sway index (r^1^ = 0.985, *P* < .001; r^2^ = 0.970, *P* < .001), total displacement (r^1^ = 0.982, *P* < .001; r^2^ = 0.935, *P* < .001), center of pressure mean velocity (r^1^ = 0.982, *P* < .001; r^2^ = 0.935, *P* < .001), ellipse radius 1 (r^1^ = 0.979, *P* < .001; r^2^ = 0.965, *P* < .001), ellipse radius 2 (r^1^ = 0.982, *P* < .001; r^2^ = 0.969, *P* < .001), and ellipse area (r^1^ = 0.983, *P* < .001; r^2^ = 0.969, *P* < .001).

**Conclusions:**

Data from this case study suggest that VRSway measurements are highly correlated with output from force plate technology posing that VRSway is a novel approach to evaluate balance measures with VR. More research is required to understand possible uses of VR-based use for balance measurement in a larger and more diverse cohort.

## 1. Introduction

Balance is the ability of the body to maintain the center of mass relative to the base of support, thereby resisting equilibrium changes.^[1]^ Maintaining balance is achieved by the complex integration and coordination of multiple systems including the vestibular, visual, auditory, motor, and higher-level premotor systems.^[2,3]^ Falls are the second leading cause of unintentional injury deaths globally,^[4]^ and falling and the inability to maintain balance are major risk factors for adults age ≥60, who suffer the greatest number of fatal falls annually.^[4]^ A variety of chronic and acute conditions are also characterized by balance difficulties, including neurological diseases and sport injuries.^[5]^ Methods to monitor and quantify balance are therefore critical for clinical decision-making regarding risk management and balance rehabilitation.^[2,3]^

The current gold standard for accurate balance measurement are force platforms. Utilizing force measurement technology, force platforms measure ground reaction forces to calculate force development and center of pressure (CoP) positioning.^[6]^ This reflects the neuromuscular response to movements in the center of gravity, and thus, closely approximates the center of gravity in slow-moving or static conditions.^[7]^ While this technology undoubtedly provides accurate and consistent force measurements, there are several drawbacks. Specifically, force platforms are expensive to purchase, require frequent calibration, and substantial maintenance. In addition, a significant amount of space is required, limiting accessibility for some research, and clinical facilities.^[8]^

New advances in virtual reality (VR) technology has identified VR as a novel therapeutic platform. Most VR devices consist of a wearable 3D display device comprising a pair of glasses and headphones that are connected to a computer or cell phone. Some VR systems also have accelerometers, which are sensors that quantitatively measure static and dynamic acceleration in 3D and are common components in digital platforms that require human locomotion monitoring. Thus, VR systems provide computer-generated input to multisensory, interactive 3-dimensional (3D), unique experiences that can be considered a therapeutic distraction. Relatedly, VR systems are under investigation for management of anxiety and pain for patients undergoing certain medical treatments^[9–14]^ and for stroke rehabilitation therapy.^[15–17]^ Similarly, and in relation to the current case study, VR has been successfully utilized for gait and balance training/rehabilitation.^[18,19]^ Tri-axial accelerometers are a key feature of VR hardware, which has been previously used to analyze, monitor and affect postural stability.^[20,21]^ Moreover, the VRSway software was developed with the intention to be used in a variety of active rehabilitation applications, with the Sway balance mobile application most recently having been validated against the force platform technology for balance measurements.^[20]^ This case report evaluates balance measures in 2 healthy participants with no previous history of balance disorders using the VRSway software application and compares to output generated by force platform technology. Specifically, VRSway is a VR application that uses sensors attached to a virtual reality headset, and hands remote controllers for measurement and analysis of postural stability by measuring changes in spatial location relative to the center of mass and calculates various postural stability indexes.

## 2. Case presentation

The primary objective of this case study was to demonstrate the feasibility of the VRSway stability score for evaluation of balance. For this reason, participants with preexisting symptoms overlapping with major symptoms of cyber-sickness such as headache, vertigo, ataxia, nausea, vomiting or any neurological disorders that can impact balance were excluded from this case study. Here, we present posturograpy measures of the VRSway in comparison with force plate readouts in 2 healthy participants, 1 28-years old male and 1 29-years old female, with no history of any conditions that can affect stability or their ability to use a VR environment. Both participants provided informed consent. Study staff assistance was provided to participants with placement of the VR equipment. This included a VR head piece referred to as the head mounted display (HMD), a right hand sensor and left hand sensor. The VR hardware is connected to a computer capable running VRSway software for balance assessment output. Both participants were unmarried and single, spoke Hebrew, went to the Academy, and are Jewish. This study was conducted at the Center for Advanced Technologies in Rehabilitation of Sheba Medical Center and was approved by the Institutional Review Board of Sheba Medical Center.

**Figure 1.**
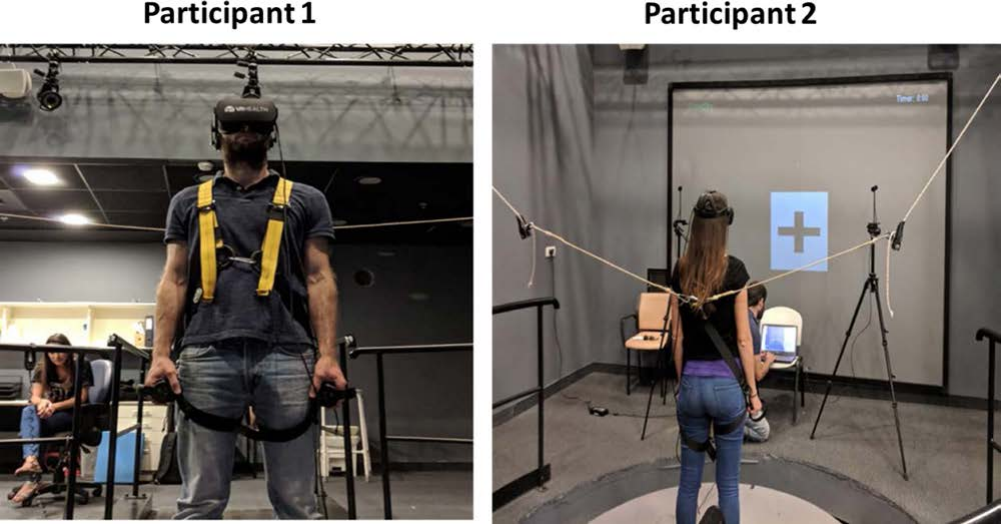
Participant placement during assessments.

In total, participants performed 30 measurements under 6 conditions, which comprised the 3 systems involved in balance: visual, vestibular, and sensorimotor. For each condition, participants were asked to stand still on the force plate with legs pelvic width apart, hip joint in neutral position, and feet parallel (Fig. 1). Body Sway measurements were recorded simultaneously in both Advanced Mechanical Technology Inc, (AMTI, model OR6-7) and VRSway systems under the 6 conditions described in Table 1.

**Table 1:** Investigational conditions.

Condition		Objective
1	Eyes open on a firm surface: baseline	Incorporates visual, vestibular, and somatosensory inputs
2	Eyes closed on a firm surface: eliminate visual input	Evaluate vestibular and somatosensory inputs
3	Eyes open on a dynamic surface: baseline	Evaluate somatosensory interaction with visually input
4	Eyes closed on a dynamic surface: eliminate visual input	Evaluate somatosensory interaction with vestibular input
5	Visual conflict on a firm surface: some vision present but information conflicts with vestibular information	This condition brings in more vestibular and somatosensory inputs
6	Visual conflict on a dynamic surface: some vision present but information conflicts with vestibular information.	Evaluate the mediation of visual with and without vestibular and somatosensory inputs

Participants were asked to perform 2 sets of tests. The first set of tests included conditions 1 to 4 (detailed above), which was performed first on a stable surface and then on a dynamic unstable surface (Airex Elite Balance Pad, Switzerland). The duration of each test is 30 seconds with a 1-minute break between each test. The second set of tests included conditions 5 and 6, which incorporated a visual conflict presented in the VR environment. As before, the participants were required to perform the test on a stable surface for 30 seconds and then on a dynamic surface for a duration of 30 seconds, but this time there was a 2-minute break between each test. Investigational conditions 1, 2, and 5 were measured 6 times for each participant, while conditions 3, 4, and 6 were measured 4 times for each participant.

**Figure 2.**
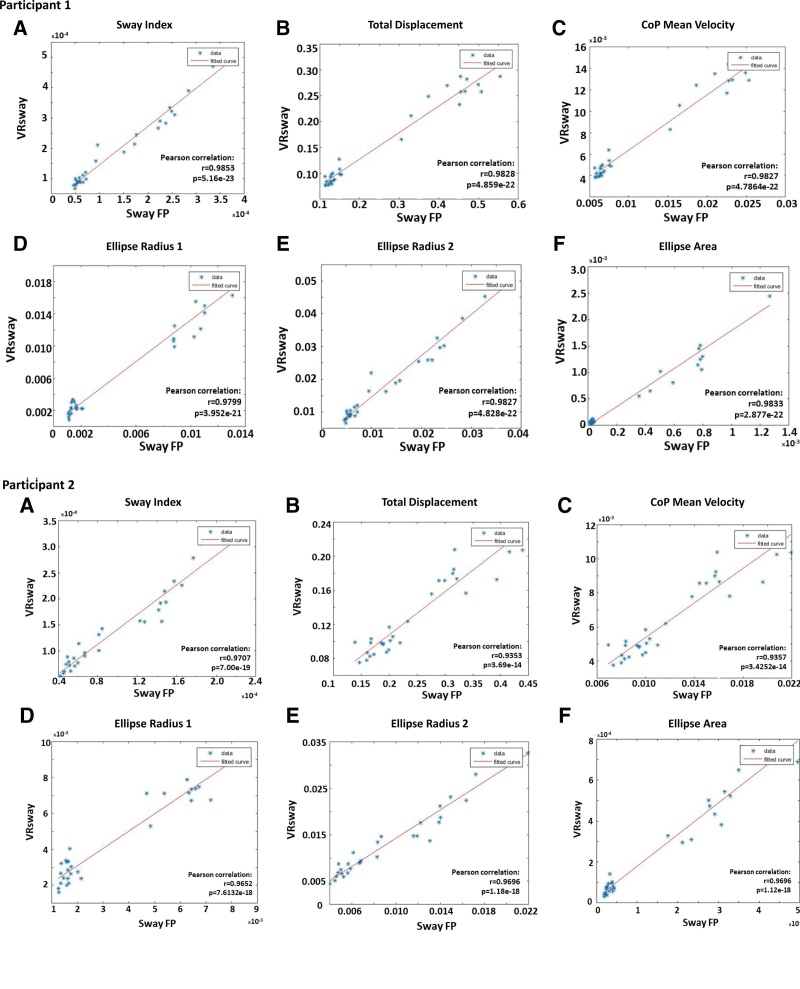
Correlations between VRSway and Sway force plate balance measurements for participant 1 and participant 2. FP = force plate, VR = virtual reality.

The VRSway results were calculated from the VR system HMD) with 6 degrees of freedom raw data (X, Y, Z, Roll, Pitch, Yaw) sampled in 90Hz, recorded from the system HMD, data from right hand sensor and left hand sensor were collected but not used for the analysis. The gold standard data were collected from AMTI force plate which generates orthogonal raw data (X, Y, Z) in 120HZ. Both VRSway and AMTI force plate output was synthesized using MTALAB 2016b. For each condition, the Sway index was calculated using an algorithm developed by XR Health specifically for the VRSway software. A correlation between the 2 balance measurement systems was calculated for 6 different Sway indexes: total displacement, CoP mean velocity, ellipse radius 1, ellipse radius 2, and ellipse area measures.^[6,22–24]^ A Pearson correlation coefficient was calculated for each participant, for all measurements according to standard practice (Table 2, Fig. 2).

**Table 2:** Correlations between VRSway and force plate for balance measures.

Balance measure	Participant 1		Participant 2	
r	*P* value	r	*P* value
Sway index	0.985251	5.16E–23	0.9707	7.00E–19
Total displacement	0.982669	4.86E–22	0.9353	3.69E–14
CoP mean velocity	0.982688	4.79E–22	0.9356	3.43E–14
Ellipse radius 1	0.979845	3.95E–21	0.9651	7.61E–18
Ellipse radius 2	0.982677	4.83E–22	0.9695	1.18E–18
Ellipse area	0.983311	2.88E–22	0.9696	1.12E–18

CoP = center of pressure, r = pearson correlation coefficient, VR = virtual reality.

## 3. Discussion

This case report demonstrates results calculated by proprietary software is highly correlative to the measurements generated by force plates, which is currently the gold standard of balance measurement. Of note, the correlation coefficients for all 6-tested measurement indexes indicate a strong level of predictability between the 2 balance measurement platforms. Given this is a small case study of 2 healthy participants, the next phase for this research requires validation of these results in a larger cohort including both healthy participants and participants with balance disorders. This will provide more insight into how VR balance measurement can provide gains in mobility, functional independence and quality of life to a variety of individuals. While the current data surrounding VR-based therapies and rehabilitation remains controversial, there is mounting evidence to support combining conventional based-treatments with VR-augmented therapies improve patient outcomes.^[17,25–28]^ However, the fact that VR platforms can provide treatment without leaving the comfort of your home a major advantage in providing medical care to patients in more remote, less accessible areas of the world and takes the concept of telehealth to the next level. Given that falls are a major leading cause of unintentional injury deaths globally, particularly in individual ≥60 years of age, there is a large area of unmet need VRSway technology may be able to fulfill as both a potential VR-based balance therapy or a preventative medical application.^[18]^

In addition to being highly correlative to force plate balance measurements, VRSway technology is less expensive to implement. Specially, the training, set-up and maintenance of the current force plate platforms requires specialized personnel to install, calibrate and maintain. The force plates also require a significant sized footprint, which limits the number of research facilities and medical centers that are able to accommodate such a system. VR-based technology on the other hand, require little space and as a result becoming commonplace in medical settings. This technology does not require an official install, offers remote training and infrequent, user-friendly calibration that does not require specialized personnel. Another advantage of VRSway technology is the ability to create a visual conflict inside the VR platform by defining only the parameters of the visual conflict, such as adjusting how fast the screen moves up-down-right-left and for how long. Up until recently, visual conflict has only been able to be inserted with heavy and expensive machines.^[29–32]^

A limitation of this method for balance measurement is the learning curve of the VR headset, particularly for those not comfortable with new technology. Other common VR limitations such as motion sickness, eye fatigue, disorientation, nausea and neurologic conditions like epilepsy are also necessary to take into consideration when implementing this method for balance measurement. However, with upfront training and educating of study staff, participants, patients and caregivers on how to access and utilize the VR headset, user difficulty can minimize the learning curve. Moreover, training of the VRSway platform for larger scale use requires less time, specialized personnel, calibration and maintenance than the current gold standard force plate platform. In addition, the VRSway platform may allow for greater accessibility across clinical specialties and expand the abilities to identify specific patient populations that may benefit from balance interventions and therapies. Taken together, data from this case study identifies VRSway as a novel approach to evaluating balance with VR and more research is required to understand possible uses of VR-based use for balance measurement in a larger and more diverse cohort.

## Acknowledgements

JetPub Scientific Communications, LLC supported by funding from XR Health, provided drafts and editorial assistance to the author during preparation of this manuscript.

## Author contributions

**Conceptualization:** Omer Weissberger, Eran Orr, Shani Kimel-Naor, Meir Plotnik.

**Data curation:** Omer Weissberger, Miki Levy, Shani Kimel-Naor, Tal Arbel.

**Formal analysis:** Omer Weissberger, Shani Kimel-Naor, Meir Plotnik, Tal Arbel.

**Investigation:** Omer Weissberger.

**Methodology:** Meir Plotnik.

**Writing – original draft:** Omer Weissberger, Shani Kimel-Naor.

**Writing – review & editing:** Omer Weissberger, Eran Orr, Shani Kimel-Naor, Meir Plotnik.
